# Acoustic Force-Based Cell–Matrix Avidity Measurement in High Throughput

**DOI:** 10.3390/bios13010095

**Published:** 2023-01-06

**Authors:** Yao Wang, Jasmine Jin, Haoqing Jerry Wang, Lining Arnold Ju

**Affiliations:** 1 School of Biomedical Engineering, The University of Sydney, Darlington, NSW 2008, Australia; 2 Charles Perkins Centre, The University of Sydney, Camperdown, NSW 2006, Australia; 3 The University of Sydney Nano Institute (Sydney Nano), The University of Sydney, Camperdown, NSW 2006, Australia

**Keywords:** extracellular matrix, mechanobiology, acoustic force spectroscopy, integrin

## Abstract

Cancer cells interacting with the extracellular matrix (ECM) in the tumor microenvironment is pivotal for tumorigenesis, invasion, and metastasis. Cell–ECM adhesion has been intensively studied in cancer biology in the past decades to understand the molecular mechanisms underlying the adhesion events and extracellular mechanosensing, as well as develop therapeutic strategies targeting the cell adhesion molecules. Many methods have been established to measure the cell–ECM adhesion strength and correlate it with the metastatic potential of certain cancer types. However, those approaches are either low throughput, not quantitative, or with poor sensitivity and reproducibility. Herein, we developed a novel acoustic force spectroscopy based method to quantify the cell–ECM adhesion strength during adhesion maturation process using the emerging z-Movi^®^ technology. This can be served as a fast, simple, and high-throughput platform for functional assessment of cell adhesion molecules in a highly predictive and reproducible manner.

## 1. Introduction

Cancer metastasis, where malignant cells spread from a primary site to distant organs in the body, is largely responsible for the mortality and morbidity of cancer [1]. After dissociating from the original tumor mass, metastatic cells migrate through the extracellular matrix (ECM) by reorganizing their attachment to the ECM with altered cell–ECM adhesion dynamics, and then invade through adjacent tissues and intravasate into the blood vessels [2]. These motile cells journey through the vascular circulation and extravasate via invading the vascular basement membrane and ECM again to ultimately attach at a new location and grow into the secondary tumor [2]. Therefore, tumor cells experience alterations in cell–cell and cell–ECM adhesion during various stages of cancer invasion and metastasis.

In the tumor microenvironment, integrin receptors mediate cell adhesion with ECM ligands (such as fibronectin or FN, laminin, collagen, and gelatin) [3,4]. Such interactions couple the extracellular environment to intracellular signals via the actin cytoskeleton, which enables cell responses to external stimuli in a coordinated manner and enhances cancer cell proliferation, migration, invasion, and metastasis [2,4,5,6]. The biophysical and biochemical cues from the tumor ECM modulates each one of the ‘hallmarks of cancer’, and defective mechanosensitivity and mechano-responsiveness of the cell–ECM interactions are often associated with metastasis progression [7,8,9]. Therefore, the cell–ECM adhesion strength or avidity has been considered as a general indication for the metastatic potential of tumor cells, which defines the aggressiveness of cancer cells [6,10,11]. Many cell-adhesion proteins have been proposed as potent targets for inhibiting cancer cell invasion and metastasis [6].

Existing techniques established to measure the cell–ECM avidity include fixed-cell imaging based in vitro adhesion seeding assays [12], micropipette-based adhesion frequency assays [13,14,15], optical tweezers-based traction force microscopy [16], as well as the conventional wash assays [17]. These model systems are able to identify key adhesion components and correlate with metastatic progression [12] thereafter providing invaluable insights into regulatory mechanisms. However, those methods are either low throughput, not quantitative, or with poor sensitivity and reproducibility [11,17]. In recent years, Engler et al. established a population-based adhesion assay using the spinning-disk shear to quantify the cell adhesion strength on fibronectin and correlate it with focal adhesion assembly. This method also captured the adhesion heterogeneity within the studied cell population [10,11]. However, this measuring system is a custom-built spinning-disk device, and the experimental operation is time-consuming, which requires relatively long sample preparation and processing time with multiple experimental steps. These aspects limit its application as a high-throughput screening system.

**Figure 1:**
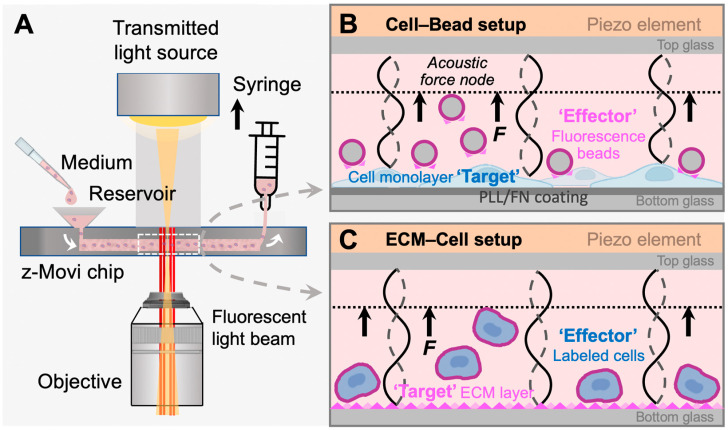
z-Movi working principle. (**A**) z-Movi utilizes a piezo-embedded microfluidic chip to perform the cell avidity measurement. The targets and effectors in the cell medium will be injected into the reservoir and then flushed into the flow chamber by pulling the syringe attached to the outlet of the chip. The light path of the imaging platform will acquire both transmitted and fluorescent light through the objective simultaneously. The target cells or extracellular matrix (ECM) proteins were flushed in first and incubated in the flow chamber to form a monolayer. To form a monolayer of cells (**B**), the chip was pre-coated with fibronectin (FN) or poly-L-lysine (PLL), which promotes cell attachment. Otherwise, the flow chamber bottom surface was coated with ECM proteins via physical absorption (**C**). (**B**) Cell–Bead setup. After coating the flow chamber bottom glass with a monolayer of target cells, fluorescence beads coated with fibronectin were flushed in as effectors and incubated with the monolayer to form interactions. Then, the piezo element driven by the software generated a standing acoustic wave in the flow chambers and applied the lifting force to the effectors to the acoustic force node (dashed line). (**C**) ECM–Cell setup. Replacing fluorescent beads in the Cell–Bead layout, labelled effector cells were flushed into the flow chamber and formed interaction with the ECM protein monolayer.

To this end, we established a user-friendly, high-throughput yet effective method to study cell–ECM interaction in vitro using the acoustic force-based z-Movi^®^ cell–cell avidity analyzer (Figure 1A). This technology allows sample preparation and avidity testing to be performed within a few hours. It can determine avidities of up to 400 cell pairs in a single run within a few minutes with minimum cell damage and high sensitivity and generate instant statistically relevant data. The z-Movi has rapidly emerged to measure the bona fide avidity between a T cell and its target tumor cell, which determines the adhesive strength of immunological synapse formation and T cell activation [18,19,20]. Assessments of the cell avidity in vitro have been proven to accurately predict cellular responses in vivo and outcomes during immunotherapy and facilitate the selection of optimal immune cells (e.g., CAR-T or TCR-T cells, NK cells) to kill cancer [18,19,20]. Here, we optimized and repurposed the z-Movi technology, for the first time, to measure cell–ECM avidity (Figure 1B,C), particularly the cell avidity with FN as a showcase. This method can be served as a rapid, simple, and high-throughput screening platform for functional assessment of cell adhesion molecules and their interactions with the tumor microenvironment in a highly predictive and reproducible manner.

## 2. Materials and Methods

### 2.1. Cell Culture

The human ovarian cancer cell line A2780 (ECACC 93112519) and human breast cancer cell line MCF-7 (ATCC HTB-22) were cultured in RPMI1640 medium (11875-119, Gibco, Waltham, MA, USA) supplemented with 10% fetal bovine serum (FBS; 10099141, Gibco), and kept in a humidified 37 °C incubator with 5% CO_2_. All cell lines were negative for mycoplasma using real-time PCR-based screening.

### 2.2. Chip Glass Surface Coating with Poly-L-Lysine (PLL)

The chip needs to be cleaned and dried for at least 1 h at 37 °C before coating. The surface coating was carried out using 0.002% PLL (P4707, Sigma-Aldrich, Macquarie Park, NSW, Australian) in PBS (10010023, Thermo Fisher Scientific, Waltham, MA, USA). For each chip, 50 μL of fresh PLL solution was prepared and pulled into the microfluidic channel using a syringe (SS+03L1, Terumo Medical Canada Inc., Vaughan, ON, Canada) with 10 μL volume left in the reservoir to avoid bubble formation. After 15 min-incubation at room temperature, the PLL solution was completely removed using the syringe. The chip was pulled through with air several times to remove any remaining liquid in the microfluidic channel. The PLL-coated chips were kept in a dry incubator at 37 °C with a cap onto the reservoir (to avoid evaporation) for at least 1 h. The coated chips must be used within 3 days after coating. Upon cell seeding, the chips were rehydrated by pulling in 100 μL warm PBS into the microfluidic channel, leaving about 10 μL in the reservoir to avoid bubble formation. This step was repeated once with 100 μL warm complete cell culture medium.

### 2.3. Chip Glass Surface Coating with Fibronectin

The chip needs to be cleaned and dried for at least 1 h at 37 °C. For each chip, the glass surface was rehydrated by pulling in the following solutions to the microfluidic channel using a syringe (leave about 10 μL in the reservoir to avoid bubble formation): 200 μL 1M NaOH (incubation for 5 min), 400 μL Milli-Q water for two times, 400 μL 1 M HCl for two times (incubation for 2 min at the second time), 400 μL Milli-Q water for three times, and 400 μL PBS once. For each chip, 50 μL of 100 μg/mL fresh FN (F0895, Sigma-Aldrich) solution in PBS was pulled into the microfluidic channel with about 10 μL left in the reservoir. The inlet was washed with 400 μL PBS three times to prevent fiber or aggregate formation and re-filled with 200 μL PBS. Chips were caped firmly and kept in the 37 °C dry incubator overnight. Before cell seeding, the FN-coated chips were washed 3 times with PBS and once with the complete culture medium. The coated chips need to be used within 3 days after coating.

### 2.4. Target Cell Monolayer Formation

Target cells with at least 80% confluency were used for z-Movi experiments. Adherent cells were washed once with PBS and trypsinized using TrypLE (12605010, Thermo Fisher Scientific) for 3–5 min at 37 °C. Cells were collected using the complete culture medium, and the cell density was determined using the automatic cell counter. For each chip, 20 μL of MCF-7 or A2780 cells at indicated densities were seeded into the microfluidic channel using a syringe (leave about 10 μL volume in the reservoir to avoid bubble formation). It is important to keep the chips and cells at 37 °C during cell seeding to reduce cell clumping. The inlet was washed with 400 μL complete medium three times and re-filled with 400 μL PBS. Chips were caped firmly and kept in the 37 °C dry incubator for a designated period (1-4 h, cell-line dependent) before z-Movi avidity assay.

### 2.5. Cell Viability Measurement of the Target Cell Monolayer

The viability of the cell monolayer was evaluated by adding 10 μL Trypan Blue (15250061, Thermo Fisher) in the reservoir containing 50 μL medium. The Trypan Blue dilution was pulled into the microfluidic channel and incubated with the monolayer cells for 30 s to stain the dead cell population, followed by 200 μL complete culture medium to rinse out Trypan Blue. The cell viability was observed by brightfield imaging.

### 2.6. Effector Cell Staining

Adherent cells were washed once with PBS and trypsinized using TrypLE for 3−5 min at 37 °C. Cells were collected using the complete culture medium, and the cell density was determined using the automatic cell counter. For a single run on the chip, 20 μL of 15 × 10^6^ cells/mL (0.3 × 10^6^ cells) was prepared. Cells were washed once with PBS and stained with the 1× CellTrace Far Red Dye (C34564, Thermo Fisher) dilution in PBS at 1 × 10^6^ cells/mL in the dark at 37 °C for 15 min. Cells were re-suspended by pipetting every 5 min during the staining process. Approximal 5 mL complete culture medium was then added to the cell solution to stop the staining. Stained cells were washed once with PBS, re-suspended in complete culture medium at 15 × 10^6^ cells/mL, and transferred into a 96-well plate with round bottom.

### 2.7. Effector Beads Coating with Fibronectin (FN)

The red-fluorescent melamine resin particles (MF-FluoRed-L840, microParticles GmbH, Berlin-Adlershof, Germany) were washed with 500 μL PBS three times and once with 500 μL complete culture medium. Beads were then incubated with FN at 10 μg/mL in 200 μL of completed culture medium for 1 h on a rotating stage at room temperature. FN-coated beads were washed three times with 500 μL complete medium and re-suspended in the complete medium (10 μL for a single run).

### 2.8. Cell Binding Avidity Measurement

The chip was placed on the z-Movi (LUMICKS B.V., Amsterdam, The Netherlands) stage, and the in-chip cell monolayer quality was evaluated and validated by applying force at 1000 pN for 10 s. Once the monolayer passed the validation, the medium level in the inlet was brought down to about 10 μL. 20 μL of stained effector cells were added into the reservoir and pulled into the microfluidic channel to interact with the target cell monolayer for a designated period. Depending on the effector density, up to 400 effectors that being considered as individual events will be incubated in the field of view (FOV) for the avidity measurement. During the incubation, the inlet was gently washed with complete culture medium three times to remove the remaining cells and refilled with 100 μL complete medium. After interaction, the acoustic force was applied with a linear force ramp from 0 to 1000 pN over 2.5 min. The percentage of bound cells to the monolayer under different levels of applied force was calculated simultaneously. To block FN binding with cancer cells, a FN antibody (MA5-11981, Thermo Fisher) was used. To block non-specific bindings, 2% BSA (A3311, Sigma-Aldrich) in complete culture medium was used.

### 2.9. Chip Cleaning

The cleaning solution containing 5% bleach (A1727, Sigma-Aldrich) was pulled into the microfluidic channel, and the chip was incubated at room temperature for at least 20 min and up to 4 h. The channel was then washed with 400 μL Milli-Q water and the glass surface was scrubbed by introducing air and moving the bubbles back and forth several times. The chip surface was scrubbed with 200 μL bleach twice, followed by pulling through (1) 400 μL bleach, (2) 400 μL Milli-Q water twice, (3) 100 μL 12M HCl (H1758, Sigma-Aldrich), and (4) 200 μL Milli-Q water twice. The surface was then scrubbed with 400 μL 1 M NaOH twice and incubated with 1 M NaOH for 1 h. After pulling through the NaOH solution, the surface was scrubbed with 200 μL 1 M NaOH twice, followed by washing with 400 μL Milli-Q water twice. Cleaned chips were stored in the dry incubator. 

### 2.10. Avidity Data Analysis

**Figure 2:**
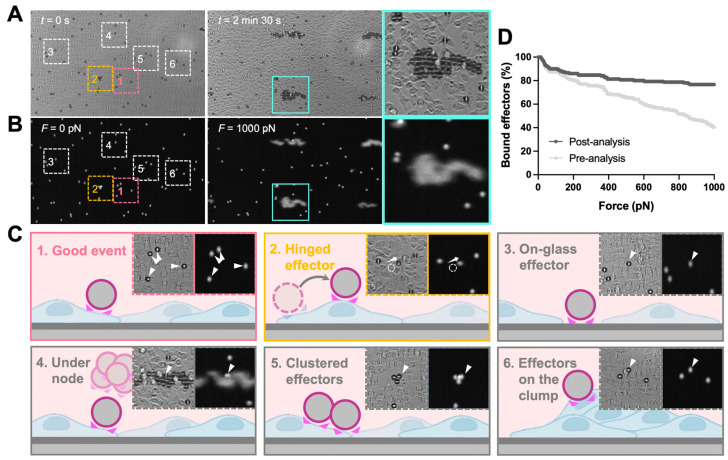
z-Movi image analysis and data selection criteria. z-Movi experiments acquired brightfield (**A**) and fluorescence (**B**) images during the force application. The brightfield images were used to examine the confluency of the monolayer and guide the manual selection. The fluorescence images were used to track the effectors whether being lifted. Zoom-ins were the representative snapshots of selected cases. Of note, if the effector escaped from the region of interest (ROI) but was not lifted during the force application (hinged, 2), the selected effector was considered as attached. Additionally, when the effectors were located on the glass surface (3), under the force node (4), clustered (5), or laid on the target cell clumps (6), these events were excluded from the analysis. Besides the listed conditions, the event was considered as a good event (1) for further analysis. (**C**) During experiments, the z-Movi applied a constant acoustic force ramping, increasing the lifting force from 0–1000 pN in 2.5 min. The lifted effectors accumulated at the acoustic force node. (**D**) Comparison between Bound cells (%) vs. Force (pN) before and after the manual selection was calculated and shown. A significant difference was seen after applying manual selection criteria. Thereby, consistent manual selection criteria are required across all runs during analysis.

The analysis of z-Movi data was performed offline by using Oceon 1.4.1 (LUMICKS B.V., Amsterdam, The Netherlands). Two-channel images were loaded into the software: (1) brightfield images which were used to examine the monolayer confluency and screen the effector events (Figure 2A, *left*); (2) fluorescence images were used to track the position of effectors (Figure 2A, *right*) and, thus, judge whether the effector was lifted. During the z-Movi application, the control software will move the field of view (FOV) to the force-calibrated region of the chip based on the information stored in the chip, while the software will capture both brightfield (monolayer tracking) and fluorescence (effectors tracking) signals within the FOV for the whole force application. The region of interest (ROI) for each effector is a 12 pixel-wide circle (with the cell as the center) by default. ROIs of up to 400 effectors within the FOV were identified by the software. Effectors left the ROIs while force application would be considered as ‘lifted’ and the software tracked all ROIs automatically. Manual selections were performed after software ROI selections (Figure 2B). Clustered effectors, effectors stuck on the glass, on the target cell clump, and under the acoustic force nodes were excluded from the analysis. In addition, effectors that escaped from the ROI but were not lifted (hinged effectors) were considered as attached effectors for the whole force application. After the manual selections, the software ran the automatic detection on lifted cells over the force ramp from 0–1000 pN (Figure 2C). Since the avidity curve would be dramatically different if no manual selection were performed (Figure 2D), we strongly recommended consistent manual selection over different runs of experiments.

### 2.11. Statistical Analysis

Unpaired two-tailed Student’s *t* test was performed in Prism 9 for the measurement of statistical significance. *p* < 0.05 was considered statistically significant. All data points with error bars are presented as mean ± standard error of the mean (S.E.M).

## 3. Results

### 3.1. Configuration of Acoustic Force-Based Measurement on Cell–ECM Adhesion

In the classic z-Movi^®^ cell–cell experimental setup (Figure 1A), the ‘Target’ cells are placed at the bottom glass of a piezo-embedded microfluidic chip. The fluorescently labeled ‘Effector’ cells are then flushed into the chip to interact with the ‘Targets’ over a certain period at 37 °C. Upon measurement, the piezo element stuck to the top glass of the microfluidic channel via a thin layer of glue generated an acoustic pulling force to ‘Effectors’. The element was driven by a function generator to excite a planar acoustic standing wave over the microfluidic channel [21], using forces ranging from 1 pN to 1000 pN to lift the ‘Effector’ to the acoustic force node. The z-Movi has a dual light path setup, allowing us to monitor the ‘Target’ with the transmitted light signal and track labelled ‘Effectors’ with the fluorescence light signal (Figure 1A). Moreover, the conversion factor for voltage amplitude in piezo to force is pre-calibrated by the manufacturer. The z-Movi^®^ software controlled the voltage amplitude supplied to the piezo element, gradually increased the force applied to the stained ‘Effectors’ and tracked their movement in the fluorescence channel. The percentage of ‘Effectors’ still bound or being lifted was enumerated over forces to indicate the level of Target–Effector avidity (Figure 2A,B,D).

In this study, repurposing the z-Movi to measure cell–ECM avidity, we developed two novel configurations to measure the cancer cell adhesion on the specific ECM component, fibronectin. For the first one, we seeded the breast cancer cells MCF-7 or ovarian cancer cells A2780 in monolayer as the ‘Targets’ and used red-fluorescent melamine resin particles coated with FN (i.e., FN-beads) as the ‘Effectors’ (*Cell–Bead setup*; Figure 1B). This Cell–Bead setup enabled us to determine the FN-dependent adhesion of tumor cells that have already spread out and formed a structural organization to mimic the late stage of firm adhesion.

In the second configuration, we formed a thin layer of FN protein as the ‘Target’ on the glass bottom and stained the MCF-7 or A2780 cells with the CellTrace Far Red Dye as the ‘Effectors’ (*ECM–Cell setup*, Figure 1C). In this ECM–Cell setup, the effector cells were in suspension when flushed into the flow chamber. Cells contacted the ECM substrate (i.e., FN) with loose attachment, followed by flattening, and cell membrane spreading over the substrate surface [22]. During this process, cells establish focal adhesions that firmly anchor on the FN matrix, and cell spreading is driven by actin polymerization and myosin contraction that push the cell membrane forward [23]. Therefore, this configuration allowed us to capture the processes of adhesion maturation from initial attachment (weak interaction during sedimentation), flattening (integrin bonding during cell attachment), to fully spreading (stable focal adhesion) [22] via increasing the incubation time for the suspended tumor cells interacting with the ECM layer.

### 3.2. Optimization of Target Cell Monolayer Formation

#### 3.2.1. Chip Surface Coating for Cell Adhesion

**Figure 3:**
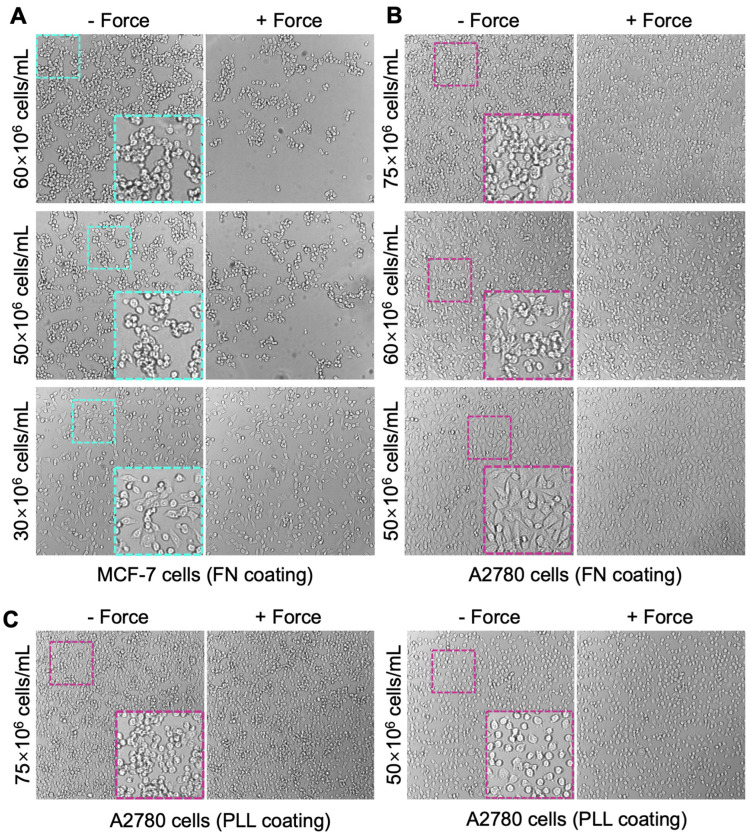
Monolayer cell seeding. MCF-7 cells (**A**) and A2780 cells (**B**) were seeded in the flow chamber at multiple density from 30 × 10^6^ to 75 × 10^6^ cells/mL to make the monolayer. The glass surface of the flow chamber was pre-coated with FN to enhance cell attachment. Representative images of the monolayer (at each seeding condition) after 2 h incubation at 37 °C (i.e., −Force) and then validation using acoustic force (i.e., +Force) were shown. After validation, weakly adhered cells were lifted from the coated surface and washed out using complete medium. (**C**) PLL coating was also tested to make A2780 cell monolayer at 75 × 10^6^ (*left*) or 50 × 10^6^ cells/mL (*right*) seeding density. Representative images of the monolayer after 2 h incubation with (+Force) or without (−Force) validation were shown. Insert: the zoom-in images showing the confluency of cell monolayer and the cell morphology.

To make a stable monolayer of solid tumor cells (i.e., MCF-7 and A2780 cells) on the glass surface of the chip, FN and poly-L-lysine (PLL) were used. Specifically, PLL enhances the electrostatic interaction between negatively charged ions of the cell membrane and the culture surface. Such a reaction caused the cells firmly attached to the chip surface after incubation for 2 h, which was validated using the acoustic force (Figure 3C). In comparison, coating with FN for 1 h has already enabled firm attachment of cells in the chip (Figure 3B), which is more efficient than PLL.

Dissociating adherent cells from the surface of plastic culturing flasks using TrypLE caused morphological changes from flattened to round shapes. After incubation on the PLL surface for 2–4 h, most cells remained round morphology with gaps between cells (Figure 3C). On the other hand, as a ubiquitous ECM glycoprotein, FN promotes cell adhesion and spreading. It largely improved the recovery of cell morphology from trypsinization. After incubation on FN for one hour, most of the cells became flattened with less empty surface space (Figure 3B), indicating a faster spreading rate as compared with that on the PLL coating. Thus, FN coating is recommended for cell seeding in the chip. 

Notably, in the Cell–Bead setup, we only included the adhesion events of FN-coated beads interacting with cells for data analysis and excluded the events where beads landed on the cell-free surface (Figure 2). Therefore, the FN (serves as ‘glue’) used for cell monolayer seeding has no impact on the adhesion measurement between tumor cells and the FN (‘Effector’) coated on the beads.

#### 3.2.2. Monolayer Cell Seeding

To generate a target cell monolayer with high confluency (at least 70%), an extremely high cell seeding density (50 × 10^6^ to 75 × 10^6^ cells/mL) is required. A2780 cells were seeded at multiple densities to determine the best condition for forming a good monolayer with minimal cell–cell gaps and maximal cell population firmly attached to the chip surface. The density of 50 × 10^6^ cells/mL produced the optimal monolayer with majority of cells spreading out and covering most of the chip surface after 1 h incubation at 37 °C (Figure 3B). Cell adhesion on the FN coating was strong enough to maintain the monolayer after the validation (Figure 3B, +Force). Increasing the seeding density to 60 × 10^6^ or 75 × 10^6^ cells/mL resulted in an obvious clumping issue, where a significant number of cell clumps formed on top of the cell monolayer (Figure 3B, −Force). The clumped cells hardly spread out even after 4 h incubation and were easily lifted by the acoustic force. However, they were hard to be removed by washing with medium due to their link with the monolayer cells that firmly adhered on the surface (Figure 3B, +Force), leading to a partial multi-layer that is not suitable for the avidity measurement.

During the preparation of suspension cell solution for monolayer seeding, some solid tumor cell lines such as MCF-7 cells are particularly prone to clumping after trypsinization. Moreover, because of the extremely high cell density required for monolayer seeding (i.e., 50–75 × 10^6^ cells/mL), the clumping issue became exacerbated. Indeed, after loading the MCF-7 cells into the chip at a density of 60 × 10^6^ or 50 × 10^6^ cells/mL, significant clumping was observed within 2–5 min, leading to large empty surfaces between clumps. Once cells clumped, they hardly spread out on the surface even with FN coating. Indeed, most of MCF-7 cells (after 2 h incubation) were lifted when the acoustic force was applied to validate the monolayer (Figure 3A, *top* and *middle*). Overnight incubation (16 h) with FN coating at 37 °C was not feasible due to the intensive cell death induced by the sealed culturing environment without CO_2_ and O_2_ exchange. Decreasing the seeding density to 30 × 10^6^ cells/mL effectively reduced the clumping rate and level (Figure 3A, *bottom*), with an increased number of MCF-7 cells spreading out and firmly attached to the chip surface. However, it also led to low seeding confluency. Although the gaps between spreading cells were small, a certain number of ‘Effectors’ will land on and interact with the surface coating (not the monolayer cells), which must be carefully identified and excluded during data analysis (Figure 2C).

**Figure 4:**
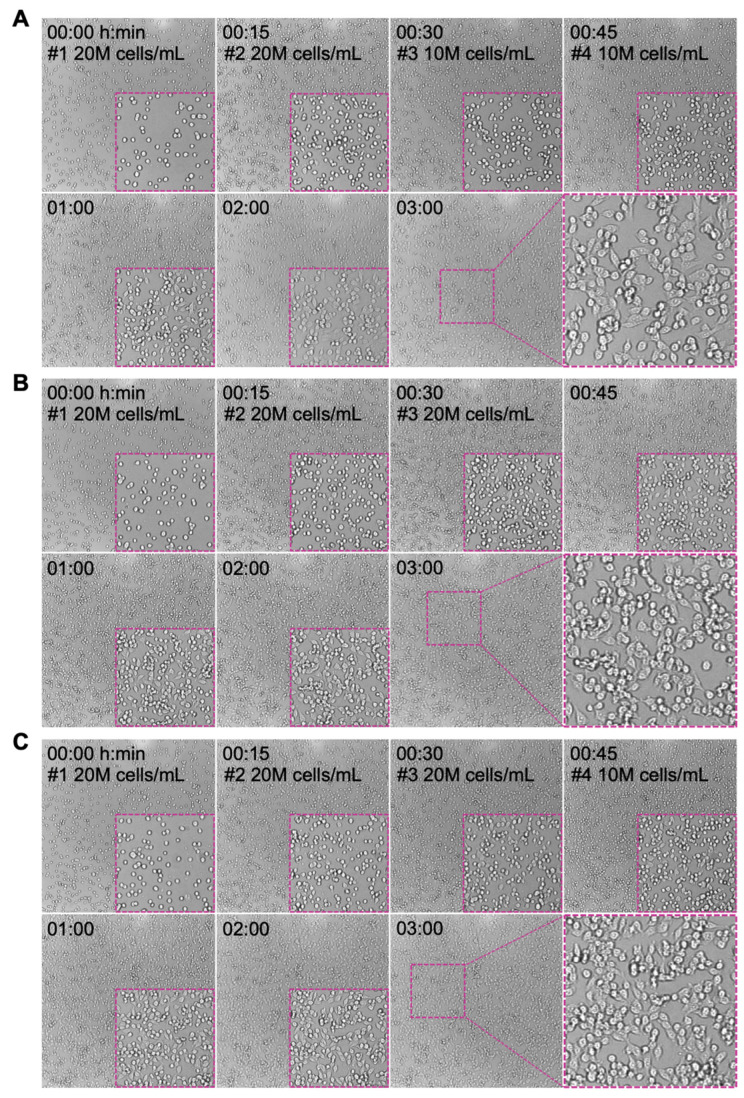
Seeding optimization to achieve target monolayer without clumpy cells. MCF-7 cells are highly clumpy at high seeding concentrations. Multiple rounds of cells seeding with low cell density were performed to avoid cell clumping. Three seeding procedures were conducted: (**A**) 20 × 10^6^ cells/mL seeding for two times followed by two times at 10 × 10^6^ cells/mL; (**B**) 20 × 10^6^ cells/mL seeding for three times; (**C**) 20 × 10^6^ cells/mL seeding for three times followed by additional seeding at 10 × 10^6^ cells/mL. The interval time was 15 min. The monolayer condition for each seeding procedures at indicated time points was imaged and presented. Insert: the zoom-in images showing the confluency of cell monolayer and the cell morphology.

To improve the seeding confluency while minimizing cell clumping, a multi-seeding strategy was conducted. MCF-7 cells at 20 × 10^6^ cells/mL density were loaded into the z-Movi chip with FN coating and incubated for 15 min at 37 °C. Cell seeding with a density of 20 × 10^6^ or 10 × 10^6^ cells/mL was then repeated one to three more times under the same condition to achieve a final density equivalent to 60 × 10^6^ (Figure 4A,B) or 70 × 10^6^ cells/mL (Figure 4C). As shown in Figure 4, the monolayer after two rounds of seeding (20 × 10^6^ + 20 × 10^6^ cells/mL) exhibited an increased confluency with similar levels of cell spreading on the surface as compared to one-time seeding at 30 × 10^6^ cells/mL (Figure 3A). Additional seeding with either 20 × 10^6^ or 10 × 10^6^ cells/mL further promote the confluency of the monolayer but with noticeable cell clumping incidence. Although the clumping level is less than the one that occurred when seeding at 50 × 10^6^ or 60 × 10^6^ cells/mL (Figure 3A), clumped cells were hardly spreading out and failed to firmly adhere on the surface, which is not suitable for avidity measurement.

### 3.3. Avidity Measurement of Tumor Cell Monolayer Interacting with ECM Proteins

**Figure 5:**
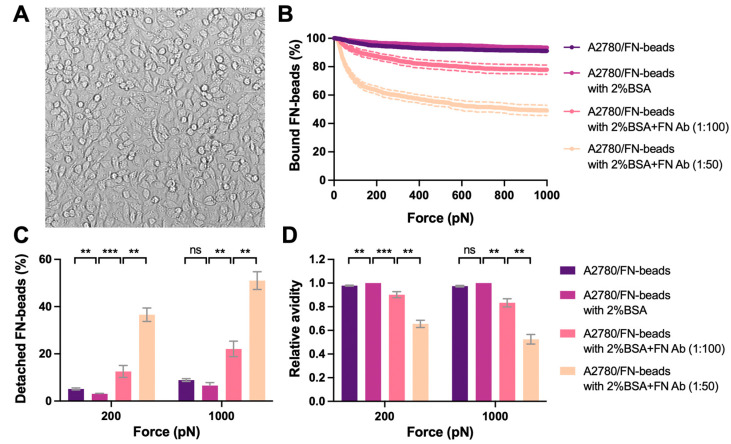
Avidity measurement of tumor cell monolayer interacting with ECM coated beads. (**A**) A2780 cells were seeded at the density of 50 × 10^6^ cells/mL. A stable monolayer was formed after 1 hr incubation at 37 °C. After validation the monolayer was used as the ‘Target’. The representative image of the monolayer was shown. FN proteins were coated on the beads as the ‘Effector’. 2% BSA was used in the complete medium to block the non-specific binding. The FN antibody was used to treat the FN-beads at 1:100 or 1:50 dilutions for 1 h to block its interaction with the ‘Target’. The ‘Effectors’ pre-treated with indicated conditions were flushed into the flow chamber and interacted with the ‘Targets’ for 2.5 min before the acoustic force was applied. (**B**) The percentage of ‘Effectors’ bound to ‘Targets’ under the force from 0 to 1000 pN was measured and analyzed to indicate the avidity. (**C**) The percentage of detached ‘Effectors’ under each treatment conditions at 200 pN and 1000 pN of force was plotted. (**D**) The avidity relative to that of A2780/FN-beads with 2% BSA was calculated and presented. Data were collected from at least three independent experiments. Error bars are mean  ±  S.E.M.; ns = not significant; ** *p* < 0.01; *** *p* < 0.001, assessed by unpaired two-tailed Student’s *t* test.

We then used the optimized monolayer of A2780 cells as proof of concept for cell–ECM avidity measurement (i.e., *Cell–Bead setup* in Figure 1B). As an intriguing matrix component found in cancer, FN was studied as a showcase. FN (10 μg/mL) was coated on the red-fluorescent beads (FN-beads) as the effectors to interact with the A2780 cell monolayer. The interaction formed rapidly after 2.5 min incubation with strong avidity, where only 3% and 6.6% FN-beads were detached from the cell monolayer under the acoustic force at 200 pN and 1000 pN, respectively (Figure 5B). It is worth mentioning that 2% BSA was added into complete medium to block the non-specific binding during avidity measurement. However, the avidity levels were similar with or without 2% BSA (Figure 5). When inhibiting FN–cell binding using an antibody specific to FN the avidity decreased in a dose-dependent manner (Figure 5C). Indeed, treatment with FN antibody (1:100 dilution) enhanced the detachment of FN-beads from 3% to 12.5% at 200 pN and 6% to 22% at 1000 pN. Increasing the concentration of FN antibody (1:50 dilution) further inhibited the interaction with 37.6% detachment at 200 pN and 51% at 1000 pN.

### 3.4. Avidity Measurement of ECM Protein Layer Interacting with Tumor Cells

**Figure 6:**
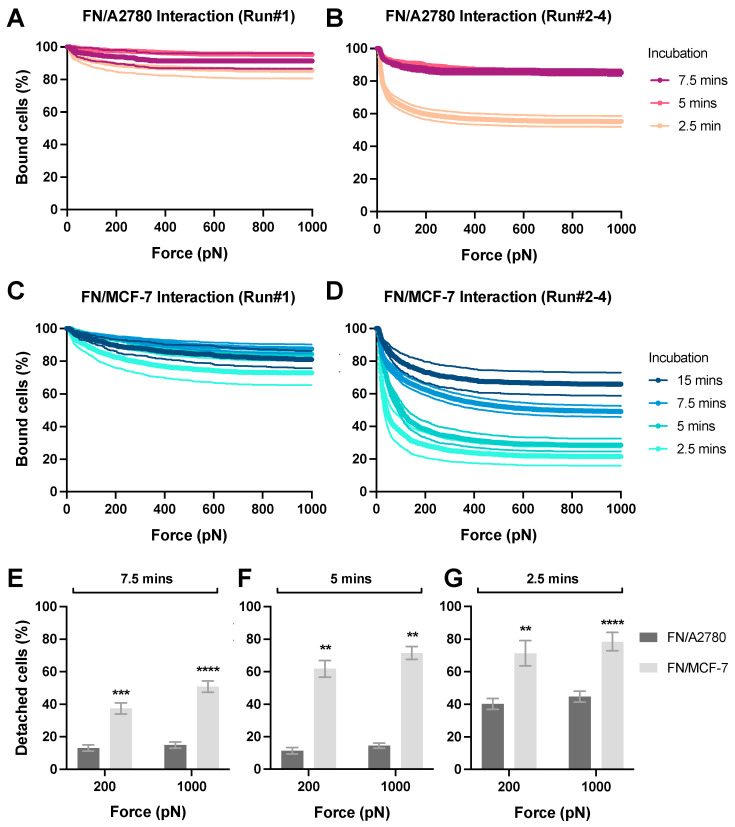
Avidity measurement of ECM protein layer interacting with tumor cells. FN protein was coated on the flow chamber surface at 50 μg/mL as the protein monolayer (‘Target’). A2780 or MCF-7 cells pre-stained with the CellTrace™ Far Red dye were flushed into the flow chamber as the ‘Effectors’ and incubated with the FN monolayer for 2.5 to 15 min before the acoustic force was applied. The percentage of effector cells bound to FN under the force from 0 to 1000 pN was measured and analyzed to indicate the avidity. The data from the first runs of each chips using (**A**) A2780 or (**C**) MCF-7 cells was shown. The data generated from the second to fourth runs in each chip using (**B**) A2780 or (**D**) MCF-7 cells was presented. After incubation for (**E**) 7.5 min, (**F**) 5 min or (**G**) 2.5 min with FN monolayer, the percentage of detached effector cells at 200 pN and 1000 pN was plotted for each cell lines. Data were collected from at least three independent experiments. Error bars are mean  ±  S.E.M.; ns = not significant; ** *p* < 0.01; *** *p* < 0.001, **** *p* < 0.0001, assessed by unpaired two-tailed Student’s *t* test.

We next performed the cell–ECM avidity measurement (Figure 1C). To visualize the effector cells during the avidity measurement, A2780 and MCF-7 cells were stained with the CellTrace™ Far Red dye. The ECM protein FN was coated on the glass surface (50 μg/mL) and incubated with stained cancer cells for 2.5 to 15 min before the avidity measurement. We observed that A2780 cells (Figure 6A) exhibited significantly higher avidity with FN than MCF-7 cells (Figure 6C). For example, after incubation for 5 min, 11–14% of A2780 cells were detached from the FN layer at 200–1000 pN (Figure 6B,F), versus 62-71% for MCF-7 cells (Figure 6D,F). A similar trend was observed with the avidity measurements after 7.5 min and 2.5 min incubation (Figure 6E,G). Moreover, to achieve the same level of avidity (e.g., 40% detached cells at 1000 pN) A2780 required a shorter interacting duration than MCF-7 (e.g., 2.6 min versus 12.5 min). Additionally, four runs of experiments were conducted for each z-Movi chip. Notably, the avidity of the Cell-ECM interaction measured during the first runs was always significantly higher than that determined via the other three runs, which produced more consistent results (Figure 6A–D). For better reproducibility, we established a protocol by excluding the data from the first runs for each chip. Taken together, these observations indicate that different cancer types exhibit substantially different cell-adhesion capacities to the ECM.

## 4. Conclusions

In brief, we have established a novel high-throughput measuring platform using the emerging acoustic force-dependent z-Movi technology to determine the adhesion strength of different types of cancer cells on ECM in vitro. This robust and highly maneuverable method will offer a rapid and simple solution to perform predictive, reproducible, and fast characterization of bona fide interactions between tumor cells and ECM components. More importantly, z-Movi allows us to capture early adhesion events and quantitate the adhesion strength from the initial cell attachment, flattening, to fully spreading on ECM, which cannot be achieved by traditional methods. Furthermore, given that acoustic force causes minimal damage to live cells, this system is suitable for studying fragile cells like primary patient samples and cancer stem cells, regardless of the tumor types. Although the experimental setup is designed for in vitro testing, the adhesion strength quantified by z-Movi serves as a general marker to predict the metastatic potential of the cancer cells *in vivo*. Moreover, by measuring the avidity of hundreds of ‘Target–Effector’ pairs in parallel with minimum cell damage with high sensitivity, this method is of great potential for preclinical drug screening to identify new candidates with potential anti-metastatic properties in a high-throughput manner.

## Data Availability

Data is contained within the article.
